# Impact of Pharmacological Inhibition of Hydrogen Sulphide Production in the SOD1G93A-ALS Mouse Model

**DOI:** 10.3390/ijms20102550

**Published:** 2019-05-24

**Authors:** Alida Spalloni, Viviana Greco, Giulia Ciriminna, Victor Corasolla Carregari, Federica Marini, Luisa Pieroni, Nicola B. Mercuri, Andrea Urbani, Patrizia Longone

**Affiliations:** 1 Department of Experimental Neuroscience, Molecular Neurobiology Unit, IRCCS Fondazione Santa Lucia, 00143 Rome, Italy; a.spalloni@hsantalucia.it (A.S.); giuliaciriminna@gmail.com (G.C.); 2 Fondazione Policlinico Universitario Agostino Gemelli IRCCS, 00168 Rome, Italy; viviana.greco@unicatt.it (V.G.); corasolla@gmail.com (V.C.C.); federica.marini@unicatt.it (F.M.); andrea.urbani@unicatt.it (A.U.); 3 Institute of Biochemistry and Clinical Biochemistry, Università Cattolica del Sacro Cuore, 00168 Rome, Italy; 4 Department of Experimental Neuroscience, Proteomics and Metabonomics Unit, IRCCS Fondazione Santa Lucia, 00143 Rome, Italy; l.pieroni@hsantalucia.it; 5 Department of Systems Medicine, Policlinico Universitario “Tor Vergata”, University of Rome “Tor Vergata”, 00133 Rome, Italy; mercurin@med.uniroma2.it; 6 Department of Experimental Neuroscience, Experimental Neurology Unit, IRCCS Fondazione Santa Lucia, 00143 Rome, Italy

**Keywords:** amyotrophic lateral sclerosis, hydrogen sulphide, pharmacology, amino-oxyacetic acid (AOA), inflammation, glial cells

## Abstract

A number of factors can trigger amyotrophic lateral sclerosis (ALS), although its precise pathogenesis is still uncertain. In a previous study done by us, poisonous liquoral levels of hydrogen sulphide (H_2_S) in sporadic ALS patients were reported. In the same study very high concentrations of H_2_S in the cerebral tissues of the familial ALS (fALS) model of the SOD1G93A mouse, were measured. The objective of this study was to test whether decreasing the levels of H_2_S in the fALS mouse could be beneficial. Amino-oxyacetic acid (AOA)—a systemic dual inhibitor of cystathionine-β-synthase and cystathionine-γ lyase (two key enzymes in the production of H_2_S)—was administered to fALS mice. AOA treatment decreased the content of H_2_S in the cerebral tissues, and the lifespan of female mice increased by approximately ten days, while disease progression in male mice was not affected. The histological evaluation of the spinal cord of the females revealed a significant increase in GFAP positivity and a significant decrease in IBA1 positivity. In conclusion, the results of the study indicate that, in the animal model, the inhibition of H_2_S production is more effective in females. The findings reinforce the need to adequately consider sex as a relevant factor in ALS.

## 1. Introduction

Amyotrophic lateral sclerosis (ALS), or Lou Gehrig’s disease, is a multisystem, progressive degeneration disorder that causes the death of the motor neurons in the spinal cord, brain stem and motor cortex [1]. The disease can be sporadic (sALS) or inherited (familial ALS, fALS) [2]. Various sex genes have been linked to ALS [3,4], spanning from control of oxidative stress (Cu–Zn superoxide dismutase, SOD1) [5], to intracellular trafficking [6,7,8], RNA metabolism (TDP-43, Fus) [9,10,11], or of a yet unclear function (C9orf72) [12,13]. Numerous mechanisms contribute to the onset and progression of the disease, including neuroinflammation [14,15]. Studies using classical ALS mouse models carrying mutations in the SOD1 gene, hereafter termed fALS, revealed that non-cell autonomous mechanisms, such as neuroinflammation resulting from the activation of glia cells like microglia and astrocytes, significantly contribute to the progression of the disease [16]. In particular, astrocytes, the most abundant glial cell type in the central nervous system, adopt a potentially neurotoxic reactive phenotype release [17] and play a key role in the pathophysiology of ALS [18,19]. In a previous study, our group found significantly elevated levels of free hydrogen sulphide (H_2_S), a toxic exogenous gas and an endogenous gasotransmitter, in the cerebral spinal fluid (CSF) of sALS patients and in the neuronal tissues of the SOD1G93A fALS mouse model, compared, respectively, to human CSF control groups and mouse WT neuronal tissues [20]. We also demonstrated [20] that glial cells are a crucial source of H_2_S in the brain. Hence, we hypothesised that H_2_S could be one of the substances released by glial cells, consequently contributing to the spreading of the disease. Further, in a more recent study [21], we demonstrated that H_2_S toxicity to motor neurons was reverted by Bax inhibitor V5 and by necrostatin, a potent necroptosis inhibitor. We also performed a proteomic analysis that revealed, under H_2_S toxicity, a significant activation of pathways related to oxidative stress and cell death. 

Along this line of research, in this study, SOD1G93A mutants were tested to determine whether treatment with aminooxyacetic acid (AOA)—a systemic inhibitor of cystathionine-β-synthase (CBS) and cystathionine-γ lyase (CSE), two key enzymes in the endogenous synthesis of H_2_S [22,23]—could be beneficial for the ALS pathology progression. Male and female SOD1G93A mice were treated with AOA starting at a pre-symptomatic stage (75 days after birth). Following treatment, it was found that AOA was able to delay the appearance of motor symptoms and slightly prolong the lifespan in the female group. It was also observed that in the spinal cord of the females, GFAP positive cells increased, while that of activated microglia decreased. However, AOA treatment did not preserve the number of spinal motor neurons.

## 2. Results

In our earlier study [20], we demonstrated an increased production of H_2_S in ALS. In this follow up study, we investigated whether systemic inhibition of H_2_S production could improve disease progression in the fALS mouse model SOD1G93A. We used AOA to prevent the generation of endogenous H_2_S. AOA, a compound known to increase brain γ-aminobutyric acid (GABA) levels [24], is also a recognised dual inhibitor of CBS and CSE that is used to arrest the production of H_2_S in the peripheral and central nervous systems [25]. The objective of this study was to assess whether a pharmacologically-induced decrease of H_2_S production could improve the outcome of ALS and whether there was a sex-specific impact of the treatment.

### 2.1. Altered Liquoral Levels of H_2_S in Male and Female ALS Patients

The demographic characteristics of the control participants and ALS subjects are described in the study by Davoli et al. [20]. As reported in that study, we measured a generalised and statistically significant increase in the concentration of H_2_S in the liquor of male and female sporadic ALS patients. A split analysis of the male and female patients highlighted that some patients in the female group had high concentrations of H_2_S (≥10 mg/L; 25% of the total sample) compared to the male group (14% of the total sample) (see Table 1), although there is not a statistically significant difference between the two groups.

### 2.2. Effects of AOA Treatment on H_2_S Production in Primary SOD1G93A Spinal Cord Culture

We know, from our previous work [20], that the spinal cord cultures prepared from SOD1G93A embryos generate H_2_S at a statistically higher rate than control cultures. In light of this evidence, we first tested the efficacy of AOA in halting H_2_S production in our ALS-mutant cultures system. AOA was administered at increasing concentrations (250 and 500 µM and 1 and 2 mM) on the same day of plating and added thereafter every other day for a week. From the 2nd to the 7th day 100 µL of medium was taken at the indicated times (see Table 2) and its H_2_S content was measured. Table 2 shows the effects that increasing concentrations of AOA have on the H_2_S levels in the culture media, levels that were reduced to a not detectable amount (ND) by our experimental and instrumental conditions. Moreover, AOA at a concentration of 250 µM was able to partially protect motor neurons (identified as SMI32+ neurons) from H_2_S toxicity (200 µM) (see Supplementary Figure S1). 

### 2.3. Effect of AOA Treatment on Motor Dysfunction, Body Weight and Survival of SOD1G93A Mice

We then investigated whether a systemic drop in H_2_S levels had a therapeutic impact on the course of the disease of the fALS mouse SOD1G93A. We started an AOA chronic treatment (8,75mg/kg/day) [26] before the onset of disease symptoms (75 days after birth). Given the differences by sex that were observed in human patients and fALS mice [27,28] and the slight difference in H_2_S levels that were measured in female patients compared to males (Table 1) [20], both males and females were treated.

**Figure 1:**
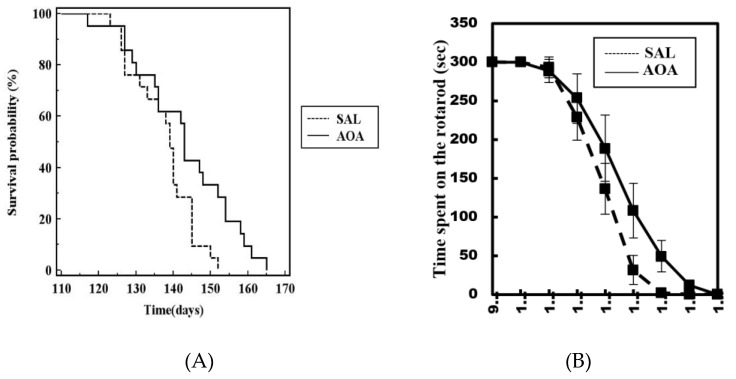
Combined Kaplan–Meier survival curve of male and female SOD1G93A. The survival curve shows a tendency toward an increase in the life span in the female group. Survival curves are not statistically significant (**A**). Treatment with AOA also had no significant effects on the rotarod test (**B**).

**Figure 2:**
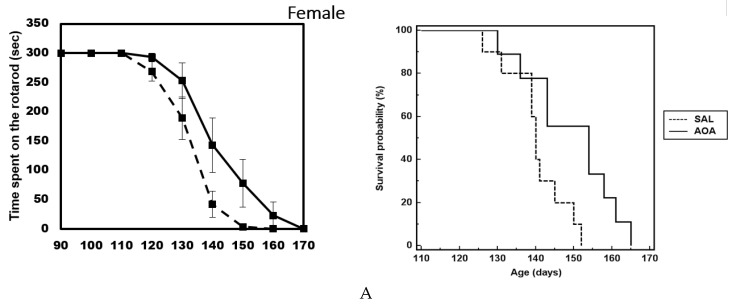

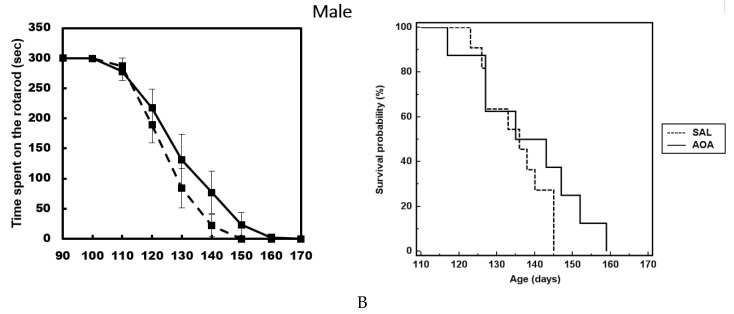
AOA was administered from postnatal day 75 to end-stage. (**A**) Median survival curve in Figure 1 treated with saline (140 days, *n* = 10) compared to females treated with AOA (154 days, *n* = 9). (**B**) Median survival curve in male SOD1G93A treated with saline (136 days, *n* = 11) compared to mice treated with AOA (139 days, *n* = 8). Survival curves are not significantly different. The figure also shows the time spent on the rotarod by the female (**A**) and male (**B**) groups.

Overall, the lack of effect is evidenced by the overlapping Kaplan–Meyer curves (Figure 1A) and rotarod curve (Figure 1B), when both groups (male and female) were combined. However, when the animals were separated by sex, we observed an effect on the rotarod performance (Figure 2A) and an increase in the lifespan of about ten (10) days of the female group compared to the male group (Figure 2B). The analysis by the Kaplan–Meier survival curve shows a tendency toward an increase in the life span in the female group (χ^2^ = 5.5948) but not in the male group (χ^2^ = 1.4858). The analysis by a two-way ANOVA, followed by the post-hoc Bonferroni’s test, shows a not significant *p* value (*p* = 0.064). The progressive loss of body weight was unaffected by the treatment in both groups (not shown). 

### 2.4. AOA Treatment Decreases the Amount of H_2_S in the Cerebral Tissues

AOA is an inhibitor of both CSE and CBS and can affect the production of H_2_S in the periphery as well as in the brain. Following the treatment, we first assessed whether AOA was indeed able to decrease the levels of H_2_S in tissues. From our previous work [20] and from published data [29,30], we know that the level of H_2_S in mice brains ranges between 2 and 4 mg/L. Concentrations of H_2_S that we measured in the saline- and AOA-treated groups are presented in Table 3. The inhibitor was able to sharply decrease the amount of H_2_S in the cerebral tissues (spinal cord, brain stem, motor cortex), but not in muscles, in both male and female treated mice.

### 2.5. Effect of AOA on Spinal cord Glial Cells

In light of the ability of AOA to slightly improve the neurological impairment in the female transgenic mice tested (see Figure 2), we next analysed whether this effect was paralleled by a reduced motor neuron degeneration in the female responding group. We, therefore, counted motor neurons in treated and untreated SOD1G93A females and found that the treatment did not reduce widespread motor neuron loss (not shown). Since we [20] and others [31] have demonstrated that H_2_S is a glial product, and we know that CBS is preferentially expressed in astrocytes [32], we assessed whether its inhibition affects glial cells expression in the spinal cord.

**Figure 3:**
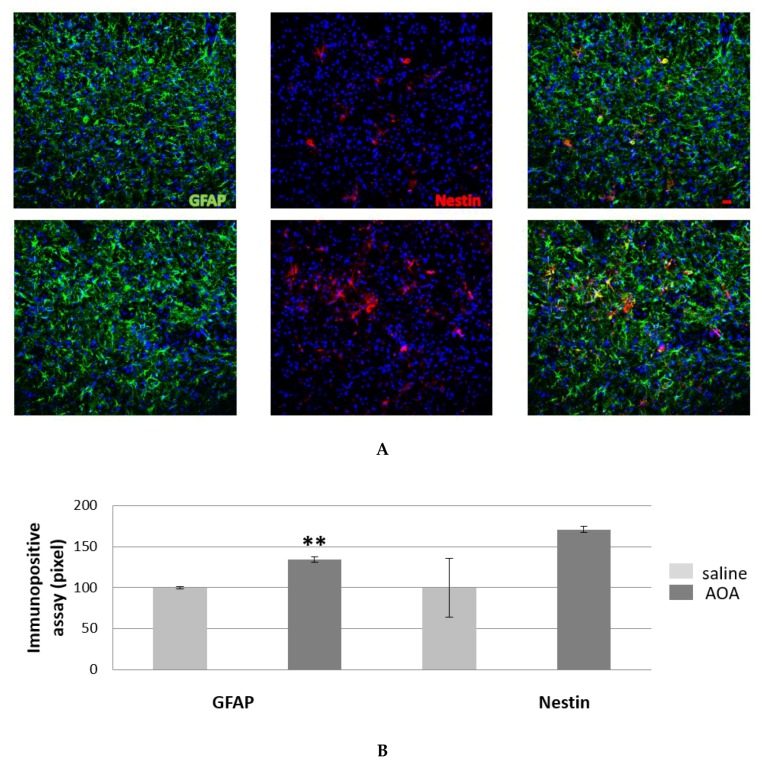
GFAP and nestin positive cells in the spinal cord of SOD1G93A saline- and AOA-treated mice. The double immunofluorescence of GFAP and nestin reveals an astrocyte increase in AOA-treated mice (bottom panel, (**A**) compared to those treated with saline (upper panel, A), also showed from immunopositive assay (**B**). The mean of the pixels was quantified in three fields of the ventral horn spinal cord in AOA-treated mice (*n* = 3) and saline-treated mice (*n* = 3), data are presented as a percentage of normalised to saline values as the mean ± SEM. The values were compared by using Student’s *t*-test with **p < 0.01 (B). Scale bar = 20 microns.

Figure 3B shows the statistically significant increase in GFAP immunoreactivity in the treated female mice compared to those who received saline treatment. GFAP is the representative marker for astrocytes and is shared by astrocyte-like subtype stem cells. We also assessed the expression of nestin, a type VI intermediate filament protein [33,34] transiently expressed in neural stem cells (NSCs) [35] and a good marker for these types of cells [36]. Nestin is known to be re-expressed by astrocytes when they respond to a central nervous system (CNS) lesion from stroke, tumour growth, or neurodegenerative diseases and become activated [37,38,39]. During their activation, astrocytes might undergo a de-differentiation process towards a multipotent lineage of neural stem cells (NSCs) [40]. Figure 3B shows that the nestin immunoreactivity was not significantly different between AOA- and saline-treated female mice, although in the AOA-treated group it showed a persistent yet not significant increment in positivity.

**Figure 4:**
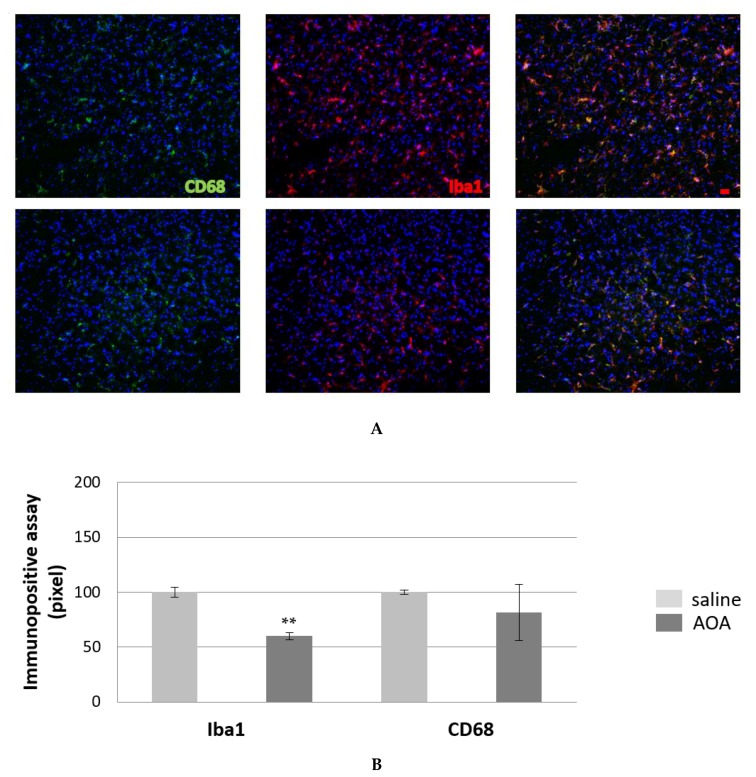
Microglia (IBA1) and reactive microglia (CD68) in the spinal cord SOD1G93A treated with saline (**A**, upper) and AOA-treated mice (**A**, bottom). (**B**) The immunopositive assay showed a decrease of microglia in AOAA-treated mice compare to those treated with saline. The mean of the pixels was quantified in three fields of the ventral horn spinal cord in AOA-treated mice (*n* = 3) and saline-treated mice (*n* = 3). Data are presented as a percentage of normalised to saline values as the mean ± SEM. The values were compared by using Student’s *t*-test with **p < 0.01 (B). Scale bar = 20 microns.

Next, we probed microglial cells with ionised calcium-binding adaptor molecule 1 (IBA1) and cluster of differentiation 68 (CD68). IBA1 is a cytoplasmic protein expressed in the monocyte lineage cells and is primarily restricted to microglia in the brain [41]. It is considered a general marker of microglia, although its expression seems to increase during microglia activation [42]. CD68 is a common marker for macrophage lineage cells, primarily localised to microglia within the brain. CD68 labels the lysosome and, because of this, it is generally considered a marker of activated phagocytic microglia [43,44]. Figure 4B shows the statistically significant decrease in IBA1 positivity in the spinal cord of the females in the AOA-treated group, while CD68 immunopositivity did not reach any statistically significant change.

## 3. Discussion

In this study, we tested whether a systemic inhibition of H_2_S production was able to improve ALS pathological features and delay their onset. Although we did not observe any significant improvement in the traits of the disease, we observed in the female group a trend toward disease amelioration and survival prolongation. A subsequent analysis of the spinal cord tissue of the female group highlighted: 1) the ability of AOA to decrease H_2_S concentration in the cerebral tissues; 2) the statistically significant increase in astrocytes positivity accompanied by a persistent increase in nestin positivity; and 3) a significant decrease in microglia positivity. The lack of clear-cut effectiveness may have different explanations: 1) the fact that H_2_S is, most likely, one of the many pro-inflammatory factors released by glial cells, and arresting its production alone is not enough for real improvement of the disease; 2) the fact that AOA is a drug with a broad pharmacology, besides being an inhibitor of CBS and CSE, it also inhibits the transamination of β-alanine and ornithine in the liver, and the transamination of GABA in the brain; 3) and, finally, the fact that ALS is a complex pathology triggered by several elements, thereby requiring pharmaco-therapies that address multiple pathological pathways simultaneously.

H_2_S, now identified as a gasotransmitter along with nitric oxide (NO) and carbon monoxide (CO) [45], has been proposed as a neuroprotectant in a number of neurodegenerative pathologies [46]. In recent years, the literature on the role of H_2_S has been inundated with conflicting claims on its effects. In inflammation, for example, it has been described as a proinflammatory agent in neutrophils [47] in burn injuries [48], and bacterial sepsis in the lung is worsened by H_2_S donors [47]. By contrast, there are numerous reports of anti-inflammatory effects of H_2_S donors in disorders of the joints [49], arthritis [50], and in severe models of colitis [51]. It is considered a cytoprotectant for neurons, as well. It can act as a potent antioxidant by stimulating the activity of the cysteine transporter and the cystine/glutamate antiporter or by increasing glutathione production and protecting the neurons against glutamate-induced oxidative stress or oxytosis [52,53]. Such contradictions reflect variations in the response to the opposing concentrations of H_2_S that might occur in the brain and elsewhere. At physiological concentrations, H_2_S is an anti-inflammatory agent but becomes pro-inflammatory at higher concentrations, a pattern similar to NO and CO, the other two gasotransmitters [45,54,55]. H_2_S has been proposed as a possible neuroprotective agent in Parkinson’s disease (PD) and Alzheimer’s disease (AD), both neurodegenerative diseases characterised by elevated oxidative stress and accumulation of misfolded proteins [56,57].

It seems that H_2_S holds this dual action in ALS, too. We measured extremely high concentration of H_2_S in sporadic ALS patients and in SOD1G93A mice [20]. We also demonstrated that in primary spinal cord cultures, at 100 and 200 µM, H_2_S is selectively toxic to SMI32+ neurons (SMI32 is used as a marker of motor neurons) but not to GABA+ neurons while, at 50 µM, it is not toxic, and at 25 µM it shows a tendency to increase the number of SMI32+ neurons [20]. These findings imply that H_2_S is likely to exert dose-dependent and cell-specific effects on ALS. Hence, we propose that the increase of H_2_S concentration is associated with and can contribute to motor neuron death and progression of the disease. In a subsequent study [21], we also demonstrated that its toxicity is via the apoptotic and necroptotic pathways.

In the present study, we investigated whether a decrease in H_2_S levels could improve the outcome of the disease in SOD1G93A mice. We used a pharmacological strategy and treated the mice with AOA, a systemic and central inhibitor of two of the major enzymes involved in the endogenous production of H_2_S, CSE, and CBS. Although AOA was able to partially protect motor neurons in culture, and dramatically decrease H_2_S concentrations in tissues, particularly in the cerebral tissues, the treatment did not improve the onset and course of the disease in any meaningful way. Nonetheless, we also observed sex dissimilarities in response to AOA treatment, with the females showing a tendency to extend the lifespan, according to the Kaplan–Meier survival curve.

Clinical observations showed sex-related differences in the etiology, progression and treatment of neurodegenerative disorders [58]. The prevalence of ALS is greater in men than in women, with a ratio of approximately 1.5:1 to 2.0:1 [28,59,60]. Moreover, a sex difference between age of onset and spinal- or bulbar-onset [61] has also been demonstrated. This is an interesting aspect, since we measured consistently higher concentrations of H_2_S in the female patients compared to the males [20] (Table 1). There is an indication that females respond better to the inhibition of production of H_2_S than males. This response could be related to the different hormonal controls, implying a slower course in females, and could be a hypothesis that explains the differences in the response to the treatment between males and females. In fact, longer exposure to female sex hormones might have a neuro-protective effect [62]. It has been reported that the average number of fertile years in women with ALS is significantly less than those without the disease which is probably due to lower exposure to cumulative estrogen [63]. We have demonstrated that treating primary spinal cord cultures with beta-estradiol resulted in the activation of the autophagic pathway, proteinaceous aggregate removal and increased cell survival [64]. In vivo, the treatment of SOD1G93A symptomatic mice with estradiol-17β resulted in improved motor performance, increased survival of motor neurons in the lumbar spinal cord and a significant drop in the expression of the NLRP3 inflammasome proteins, the levels of activated caspase 1 and of mature interleukin 1 beta [65]. Estradiol-17β can induce H_2_S biosynthesis through the stimulation of CBS and CSE mRNA and protein synthesis in the uterine artery smooth muscle in vivo [66]. Alternatively, at least in the periphery, an interaction between testosterone and the H_2_S pathway leading to a non-genomic induced vasodilatation [67,68] has been demonstrated. Hence, it seems that both male and female hormones are able to induce H_2_S production and its resulting effects. However, at this point, it is difficult to rationalise these reports with our findings.

We have shown that, in our culture system, H_2_S is released by glial cells. Therefore, it is plausible to assume that it is one of the factors contributing to the non-cell-autonomous mechanisms of MN death and that its modulation might affect the behaviour of glial cells. We observed a significant increase in GFAP positivity and a biologically significant increment in nestin positivity. Bearing in mind that the interaction between motor neurons and glial cells is pivotal in the ALS-mediated process of degeneration [16], their increase in numbers and in progenitors could be interpreted as a struggle to modify, unsuccessfully, the course of the degenerative process. Increasing evidence indicates that during CNS insults, such as neurodegeneration, astrocytes become activated, and during this process, they re-express nestin, which indicates a certain degree of de-differentiation towards a multipotent lineage [40]. Nestin is considered a useful marker for neural stem cells, its down regulation correlates with differentiation into astrocytes, neurons or oligodendrocytes. Thus, we may reason that its increased re-expression could reflect an active cellular proliferation in the treated female group as an effort to cope with degeneration. We also observed a statistically significant decrease in IBA1 positivity. This drop, generally expressed in ramified and perivascular microglia and upregulated during microglia reactive responses [42], demonstrates a generalised reduction of microglia levels. IBA1 decrease is paralleled by a decrement—although not significant—of CD68 immunopositivity. CD68 is mainly expressed in intracellular lysosomal membranes as well as in the plasma membrane. As IBA1, it is considered a general marker of microglia, and is generally upregulated. Both staining indicates an overall decrease in the microglia marker, possibly leading to a general decrease in inflammation, although this aspect will need further analysis.

In conclusion, according to our results, there has been no evidence of an effect on disease progression or life span in false mice treated with the CBS/CSE inhibitor, however, the female population appears to be more responsive. There is no doubt that different variables can contribute to this. Our results advocate for the need to deepen the investigation to develop more tailored therapies in order to effectively alter the disease progression in ALS.

## 4. Material and Methods

### 4.1. Animals

The B6SJL-TgN (SOD1-G93A)1Gur mice expressing the human G93A Cu/Zn superoxide dismutase mutation [69] were originally obtained from the Jackson Laboratories (Bar Harbor, ME, USA). Transgenic mice (SOD1G93A) and wild-type (WT) non-transgenic littermates were maintained in a C57BL6 genetic background and housed in a temperature-controlled room (22 °C) with a light–dark 12:12 cycle (lights on 07:00–19:00 h) at the Fondazione Santa Lucia-IRCCS animal facility. Screening for the presence of the human transgene was performed on tail tips of adult mice as described [70]. Food and water were given ad libitum.

This fALS mouse model reveals the initial signs of the ongoing neurodegeneration at around 90 days. At this time, mice are no longer able to mate and their body weight stops increasing when compared to the WT mice. Between 100 and 115 days, motor impairment begins and at approximately 120 days, the mice have clear difficulties in deambulation with at least one hindlimb paralysed. We selected 75 days of age (P75) as a pre-symptomatic time and the time point on when to start the pharmacological treatment. Experiments were conducted in compliance with the European Council Directive (Directive 2010/63/EU of the European Parliament and of the Council of 22 September 2010 on the protection of animals used for scientific purposes) for the use and care of laboratory animals. Every effort was made to minimise animal suffering.

### 4.2. Mixed Spinal Cord Cultures

Mixed spinal cord cultures were prepared from 14-day-old mouse embryos of a C57BL6 female mated with an SOD1G93A male as previously described [71]. Each sample was treated individually. After dissection and removal of the meninges and the dorsal horns, the spinal cords were incubated in trypsin 0.05% (Gibco–Invitrogen, Thermo Fisher Scientific Italy, 20900 Monza, Monza e Brianza, Italy) for 10 min at 37 °C. Trypsin was inactivated with fetal bovine serum (FBS, Gibco–Invitrogen), and the spinal cords were dissociated by pipetting. Each individual spinal cord was plated onto 35 mm cell culture dishes coated with poly-d-lysine (Sigma-Aldrich S.r.l., Milan, Italy) in Neurobasal medium (Gibco–Invitrogen, Thermo Fisher Scientific Italy, 20900 Monza, Monza e Brianza, Italy), supplemented with B-27, 0.5 mM glutamine and 25 μM glutamic acid.

### 4.3. Drug Treatment and Disease Progression Assessment

O-(carboxymethyl) hydroxylamine hemihydrochloride (AOA) was purchased from SIGMA-Aldrich (Sigma-Aldrich S.r.l., Merck Italia, Milan, Italy) and was freshly prepared in normal saline solution. AOA (8.75 mg/kg/day), or an equal volume of normal saline used as control, was injected intraperitoneally to male (*n* = 19) and female (*n* = 19) adult transgenic SOD1G93A mice. The mice were treated daily until the end-stage of the disease, when they were euthanised. The dosage and administration protocol were selected following Roy et al. [26].

To evaluate disease progression, starting from the 14th week of age and twice a week, mice were tested for deficit in the rotarod performance by the same operator. Body weight was also monitored. The onset of the motor symptoms was determined by the first impairment on rotarod performance for two consecutive time points. The end-stage of the disease was considered when mice were unable to right themselves within ten seconds after being placed on both sides.

### 4.4. Whole Tissue Preparation for Metabolomics Analyses and Immunofluorescence Procedures

In order to measure H_2_S levels, animals under deep anesthesia (chloral hydrate, 400 mg/kg) were decapitated and the whole spinal cords were ejected from the vertebral column, the cerebral tissues and muscles were dissected using sterile 0.1 M phosphate-buffered saline (PBS, pH 7.2). Tissues were immediately frozen on dry ice and stored at −80 °C until use.

For immunocytochemistry, treated and untreated female mice were anesthetised (chloral hydrate, 400 mg/kg) and subjected to intracardial perfusion of ice-cold 0.9% NaCl followed by 4% paraformaldehyde (PAF) in phosphate buffer (PB, 0.1 M, pH 7.2). After fixation, vertebral columns were dissected and immersed in ice-cold PAF for 16 h then spinal cords were dissected and immersed in filtered 30% sucrose/PB for cryoprotection. Spinal cords were cut on a cryostat (Leica Microsystems Srl, All Microscopy and Histology, Buccinasco (MI), 20090 Italy) to a thickness of 40 μm. For Nissl, sections were kept in 100% ethanol for 1 h and re-hydrated in descending alcohols (95% ethanol, 5 min; 70% ethanol, 5 min; 50% ethanol, 1 min) and water (3 min). The specimens were then incubated in a 0.02% cresyl violet acetate buffer solution (45 min), and the excessive stain was removed by washes in water and 50% ethanol.4.5. H_2_S Measurement by High-Performance Liquid Chromatography

As described in Davoli et al. [20], we developed specific high-performance liquid chromatography (HPLC) procedures to measure H_2_S levels in media cultures and tissues. The samples (media and tissues) were prepared according to standardised protocols described in Bailey et al. [72], with the following modifications:

Media: at the selected times, the media from the culture were treated as biological fluids. After being diluted with ionised water (1:1), a solution of 0.1N PCA (1:2) was added to the media in ice. Following vortexing and centrifugation, the samples were transferred into autosampler vials and analysed.

Tissues: following the dissection, the tissues were thawed, weighed (usually <200 mg) and sonicated in a 0.4N PCA/100 nM EDTA (1/10 *w*/*v*) solution. Following a brief centrifugation, the samples were diluted in 100 µL of mobile phase (a freshly prepared 75 mM NaOH solution, filtered, and sonicated) and transferred into autosampler vials for the analyses.

Chromatographic system: The analysis was performed on HPLC system coupled to an amperometric detector (Dionex ICS 3000; Thermo Fisher Scientific, Waltham, MA, USA), as described by Davoli et al. [20]. H_2_S measurements in samples were determined using the sodium hydrosulphide hydrate (NaHS) calibration curve prepared each time. All instrument control, data acquisition, and data analysis were performed using Chromeleon software (v6.8, Dionex, Thermo Fisher Scientific, Waltham, MA, USA). Each sample was analysed in analytical triplicate (25 µL/fraction). For CSF, samples and analysis refer to our previous work [20].

### 4.5. Immunocytochemistry

Lumbar spinal cord sections were used for double and triple immunolabelling procedures. The sections from both AOA-treated and untreated female SOD1G93A mice were rinsed in PB, and so as to subject them to identical incubation conditions, they were mounted on the same gelatin-coated slide. Sections were then processed at room temperature as follows: 5% donkey serum and 0.3% Triton for 40 min in PB, followed by 5% BSA and 0.3% Triton X-100 for 40 min in PB. After that, sections were incubated over-night at +4 °C in a mix solution of 2 or 3 primary antibodies (1% donkey serum, 0.1% X-100 Triton in PB) selected from the following: GFAP (Immunological Sciences, Società Italiana Chimici, Life Sciences, Rome, Italy; 1:500), nestin (Merck-Millipore, Merck S.p.A., Milano. Italy; 1:100), Iba-1 (FUJIFILM Wako Chemicals Europe GmbH, 41468 Neuss, Germany; 1:500), and CD68 (ABCAM, Cambridge Science Park, Cambridge, UK; 1:300). Sections were then incubated at RT with a cocktail of the appropriate secondary antibodies (Jackson ImmunoResearch Europe Ltd., Cambridge House, Cambridgeshire Business Park, Cambridgeshire, UK; 1:50). To visualise nuclei, sections were incubated for 30 min with Hoechst solution (1 ng/mL, Molecular Probes, Invitrogen, Thermo Fisher Scientific Italy, 20900 Monza, Monza e Brianza, Italy), rinsed in water and then mounted using gel mount (Biomeda Corp., Foster City, CA, USA). Controls included sections treated with secondary antibody alone and no specific staining was observed.

Sections were examined under a confocal laser scanning microscope (LSM700 Zeiss confocal microscope, Carl Zeiss S.p.A., 20156 Milano MI, Italia) equipped with four laser lines: violet diode emitting at 405 nm, argon emitting at 488 nm, helium/neon emitting at 543 nm and helium/neon emitting at 633 nm. Images were acquired in sequential scan mode by using the same acquisition parameters for all the images (laser intensities, gain photomultipliers, pinhole aperture, objective 20×, zoom 1), care was taken to avoid saturated signals in the regions of interest. Three fields of ventral horn were selected in each spinal cord (*n* = 3 for SOD1G93A and WT). To determine the degree of green and red pixels, we used the function mean pixels of ImageJ software (version 1.41n, http://rsb. info.nih.gov/ij/; National Institutes of Health, Bethesda, Maryland, USA) after background subtraction from each channel [73,74].

### 4.6. Statistical Analysis

Data were analysed and expressed as the mean ± SEM. A One-way analysis of variance (ANOVA) with a post-hoc test was used to determine whether the groups with multiple conditions were significantly different. A paired t-test was used to determine if paired groups were significantly different.

*p* values <0.05 were considered statistically significant.

The data on body weight and rotarod performance were analysed by the two-way ANOVA for repeated measures (time) and different groups (treatments), followed by a post-hoc Bonferroni’s test to compare the effect of treatments in respect to the vehicle at each time point. The analysis was applied at the time points in which all animals for each group were still alive in order to keep the number per group balanced. The age at onset and the length of survival were statistically evaluated by the Log-rank test to compare probabilities.

The data obtained from the H_2_S measurements in cell culture media and tissues (and CSF) were analysed using the Wilcoxon–Mann–Whitney test. All data are expressed as mean ±SEM, and *p* values <0.05 were considered statistically significant.

## Figures and Tables

**Table 1:** Split analyses of the H_2_S content in male and female ALS patients.

H_2_S mg/L	**ALS Total *n* = 37**	
	**Female (*n* = 16)**	**Male (*n* = 21)**
	5.52	6.38
	7.54	4.49
	**13.29**	4.60
	**13.89**	3.18
	8.44	3.49
	5.32	7.54
	4.38	6.60
	8.05	6.02
	3.86	5.04
	9.25	4.01
	7.81	9.25
	**13.40**	7.64
	8.75	**11.51**
	**10.42**	**12.39**
	2.66	8.41
	**13.34**	**10.43**
		**10.35**
		**11.30**
		5.19
		6.77
		3.12
Mean value	**8.49 ± 3.62**	**7.03 ± 2.92**

The H_2_S liquoral content in male and female patients are indicated, in bold the highest concentrations.

**Table 2:** AOA treatment inhibits H_2_S production in primary spinal cord cultures.

H2S (MG/L)	Days in Culture				
	II	IV	V	VI	VII
**NT (Not Treated)**	4.3 ± 0.4	4.5 ± 0.03	3.8 ± 0.26	3.2 ± 0.14	1.42 ± 0.16
**250 µM AOA**	2.7 ± 0.2	2 ± 0.02	1.4 ± 0.015	0.7 ± 0.21 *	ND
**500 µM AOA**	1.8 ± 0.25 *	0.9 ± 0.08 *	ND	ND	ND
**1 MM AOA**	0.4 ± 0.26 *	ND	ND	ND	ND
**2 MM AOA**	ND	ND	ND	ND	ND

Effects of the CSE/CBS inhibitor AOA on the production of H_2_S in primary spinal cord cultures. The levels of H_2_S values (mg/L) in the culture media were measured as described in Material and Methods and are presented as medians ± SE. ND = not detectable; *p < 0.05 versus the values in the NT group of the corresponding day.

**Table 3:** AOA in vivo treatment decreases H_2_S concentration in cerebral tissues.

H2S (mg/L)	G93A			
	Male	Male AOA	FEMALE	Female AOA
**Spinal Cord**	4.09 ± 0.06 ***	1.41 ±0.03	5.29 ± 0.26 ***	1.37 ± 0.021
**Brainstem**	3.71 ± 0.2 ***	1.31 ±0.02	4.00 ± 0.015 ***	1.56 ± 0.014
**Motor Cortex**	3.99 ± 0.08 ***	1.4 ± 0.15	5.00 ± 0.07 ***	1.87 ± 0.017
**Muscle**	3.60 ± 0.031	2.83 ± 0.04	4.02 ± 0.12	3.91 ± 0.012

AOA effects on H_2_S levels in cerebral and muscle tissues of SOD1G93A male and female mice. Male and female mice were treated i.p. with 8,75mg/kg/day with AOA or saline once a day, starting from the age of 75 days until the end-stage of the disease (see Materials and Methods) when they were euthanised. All cerebral tissues show a significant decrease of H_2_S (***p < 0.00001) in SOD1G93A male and female mice treated with AOA compared to those untreated. Each value is the mean ± SEM of (*n* = 6 for each group).

## Supplementary Materials

Supplementary materials can be found at https://www.mdpi.com/1422-0067/20/10/2550/s1.
