# Supplementary material for: Impact of Pharmacological Inhibition of Hydrogen Sulphide Production in the SOD1G93A-ALS Mouse Model
Source: Int J Mol Sci. 2019 May 24;20(10):2550. doi: 10.3390/ijms20102550 (PMC6567312; doi:10.3390/ijms20102550)
Supplement: Supplementary file 1 [file ijms-20-02550-s001.pdf]

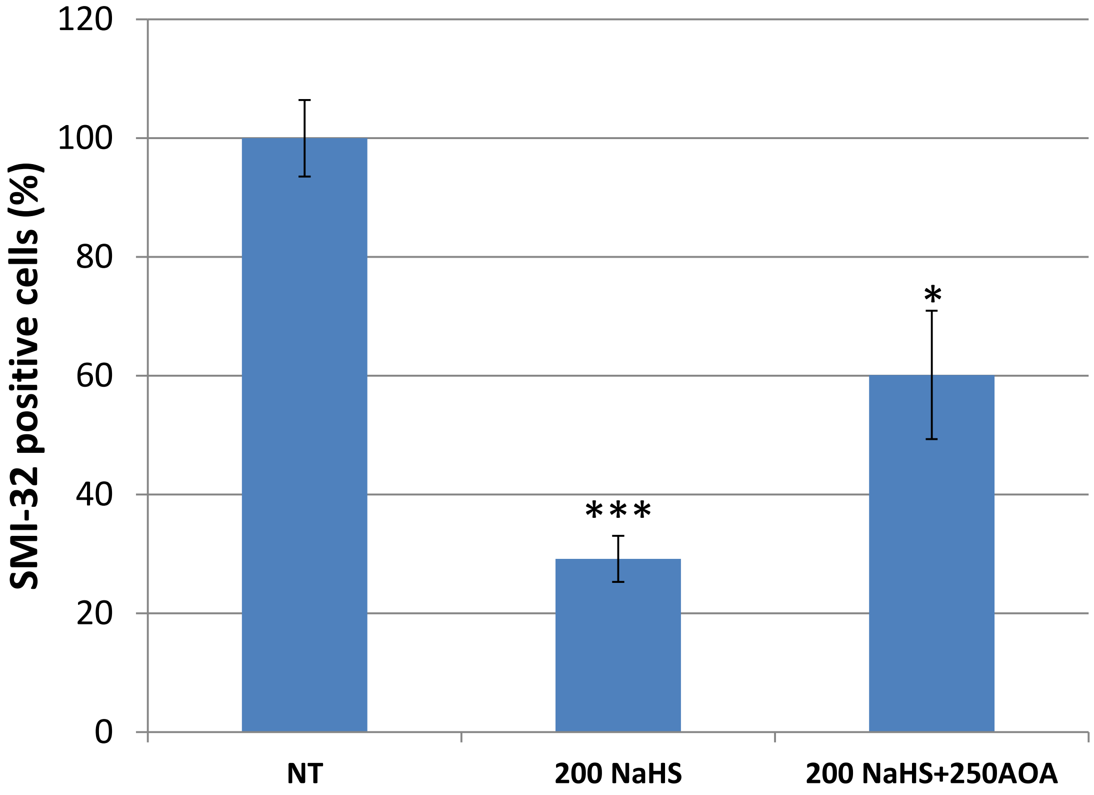

Figure S1. AOA spinal cord cultures treatment partially revert NaHS toxicity. As described in (20) and (71), the cultures at 10/12 DIV were exposed to the indicated concentrations of NaHS 200µM (n = 3) with or without 250 µM AOA (n = 3) after 18 hrs were fixed and immunostained with SMI-32. The number of surviving cells was assessed by direct counting of SMI32-positive cells and normalized to the NT values (n = 4). Each treatment was counted in triplicate. Data are presented as percentage and as mean ± SEM, and values were compared by using Student t test. ***$p < 0.005$, *$p < 0.05$ vs NT.
